# Toxicological assessment of Xanthigen^®^ nutraceutical extract combination: Mutagenicity, genotoxicity and oral toxicity

**DOI:** 10.1016/j.toxrep.2018.10.007

**Published:** 2018-10-09

**Authors:** L. López-Rios, T. Vega, R. Chirino, J.C. Jung, B. Davis, R. Pérez-Machín, J.C. Wiebe

**Affiliations:** a Nektium Pharma SL, Las Palmas, Spain; b Facultad de Ciencias de La Salud, Universidad de Las Palmas de Gran Canaria (ULPGC), Las Palmas, Spain; c P.L. Thomas & Co., Inc., NJ 07960, USA; d NOVAREX Co., Ltd. Ochang, Chungbuk, 363-885, Republic of Korea

**Keywords:** LD_50_, oral lethal dose 50%, FX, fucoxanthin, PSO, pomegranate seed oil, BSE, brown seaweed extract, SFA, saturated fatty acids, MUFA, monounsaturated fatty acids, PUFA, polyunsaturated fatty acid, NOAEL, no observed adverse effect level, DMSO, dimethyl sulfoxide, SPF, specific pathogenic free, RCC, relative cell count, MMC, mitomycin, CPA, cytophosphadine, S9, metabolic activation system consisting of liver-derived cell extract, PCE, polychromatic erythrocytes, NCE, normochromatic erythrocytes, MNPCEs, micronucleated polychromatic erythrocytes, KFDA, Korean Food and Drug Administration, Xanthigen, Functional foods, Genotoxicity, Nutraceutical, Oral toxicity

## Abstract

Xanthigen^®^ is a nutraceutical combination for weight management capable of increasing energy expenditure via uncoupling protein 1 (UCP-1) in white adipose tissue. It consists of brown seaweed *Undaria pinnatifida* extract, rich in the carotenoid fucoxanthin (FX) and pomegranate seed oil (PSO), rich in punicic acid. Xanthigen was screened to determine its genotoxicity and 90-days repeated oral toxicity. Genotoxicity was assessed with the Ames test (TA89, TA100, TA1535, TA1537, WP2), chromosomal aberration assay (Chinese hamster ovary cells) and mammalian micronucleus test (in mice). Xanthigen did not exhibit genotoxicity in any tested strain. Sub-chronic toxicity was evaluated with daily oral administration of 250, 500 and 1000 mg/kg/day doses of Xanthigen^®^ to Sprague-Dawley rats over 90 days. No deaths and no deleterious effects were observed during the 90-day treatment, indicating an absence of sub-chronic toxicity and a no observed adverse effect level greater than 1000 mg/kg/day. A statistically significant decrease in bodyweight and food intake in Xanthigen^®^ treated groups was attributed to the weight loss property of Xanthigen^®^. Overall, Xanthigen^®^ shows no significant mutagenic or toxic effects.

## Highlights

• Xanthigen^®^ is a nutraceutical combination of brown seaweed extract and pomegranate seed oil with weight management properties.
• The genotoxic potential and systemic toxicity of Xanthigen was evaluated using genetic tests and rat toxicity assays.
• Ames, in vitro chromosome aberration and mouse micronucleus test were negative.
• Xanthigen^®^ was not found to be acutely toxic, with no significant adverse effects or mortality observed at doses of up to 2000 mg/kg.
• In the subacute study, oral administration of Xanthigen^®^ for 90 days was well tolerated with a NOAEL of 1000 mg/kg/day.

## Graphical abstract

**Figure:**
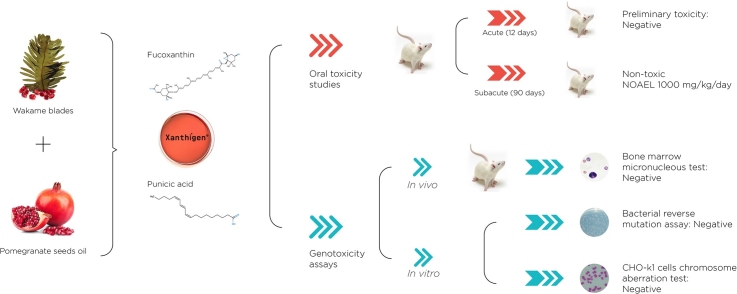

## 1 Introduction

Modern western societies are characterized by overconsumption of high-energy food and beverage combined with a sedentary lifestyle. This is associated with increasing levels of obesity and its comorbidities [1], which poses a growing therapeutic challenge, as overweight or obese individuals now outnumber leaner people in many developed and developing countries.

To tip the energy balance toward weight loss, calorie intake must be decreased relative to energy expenditure, but a reluctance to follow the minimal recommendations for an active lifestyle has created interest in safe and effective complementary natural products that can increase metabolic rate and provide an alternative to pharmacological approaches [2].

Xanthigen^®^ is a proprietary, patented combination of brown seaweed extract from *Undaria pinnatifida* (BSE) and pomegranate seed oil (PSO) standardized to ≥ 0.425% fucoxanthin (FX) and ≥ 35% punicic acid (PA).

The edible brown seaweed *Undaria pinnatifida*, traditionally known as *Wakame,* has a long history of dietary use in Asia. It is rich in proteins, vitamins and minerals, and contains the active compound fucoxanthin*, a* xanthophyll carotenoid with a unique structure that is present in several species of micro- and macroalga [3].

Fucoxanthin has been demonstrated to exert protective effects on the brain, heart, liver and skin [4] but its most unique activity is the induction of thermogenic activity in white adipose tissue (WAT) via UCP-1 activation in the mitochondrial inner cellular membrane. UCP-1 is usually expressed in brown rather than white adipose tissue. UCP-1 induction leads to fatty acid oxidation and heat production in WAT, which increases the release of energy as heat in fat tissue, thus stimulating thermogenesis. This adaptive thermogenesis plays a crucial role in energy expenditure, limiting weight gain and favoring weight loss [5].

The safety of FX is supported by a long history of traditional use and classical toxicological evaluations. The Ames test, micronucleus assay and oral dose toxicity studies performed in rat and mice have demonstrated that fucoxanthin has no mutagenic (dosage ≤ 5000 mg/plate) or genotoxic (dosage ≤ 2000 mg/plate) effects, does not increase mortality (LD50 > 2000 mg/kg) or induce abnormalities in gross appearance or in internal organs (NOAEL > 200 mg/kg/day) [4].

Punicic acid contained in pomegranate seed oil (PSO) is a powerful natural antioxidant, which has shown to possess antidiabetic, anti-obesity, neuroprotective, hypolipemic, antioxidant, anticancer and anti-inflammatory activity [6]. PSO contains a complex mixture of tocopherols, sterols, and healthy fatty acids. More than 70% of them are conjugated linolenic acids, mainly the polyunsaturated fatty acid (PUFA) punicic acid [7]. PSO has shown to be non-toxic, as no mutagenicity (Ames test; dosage ≤ 5000 mg/plate), clastogenicity (chromosome aberration test; dosage ≤ 333 μg/mL) and acute (LD50 > 5 g/kg/day) or sub chronic (NOAEL 4.3 g/kg/day) oral toxicity were observed for PSO in a safety assessment performed by Meert et al. [8] Xanthigen^®^ is consumed for its lipid-lowering effects and has been shown to induce fat loss both in rodents [9] and humans [10]. In a clinical trial conducted on 151 overweight or obese non-diabetic women who received a hypocaloric diet for 16 weeks, treatment with Xanthigen^®^ (200–1000 mg/day) resulted in a significant reduction in weight and percentage body fat without any negative effects. Further analysis of blood samples and other clinical parameters revealed statistically significant improvements in blood lipid profiles, inflammatory markers, blood pressure and waist circumference, suggesting a potential therapeutic role in the metabolic syndrome. In addition, in a 3T3-L1 cell model, Xanthigen^®^ significantly down-regulated adipogenic genes and decreased fat content in adipose tissue to a greater extent than its individual components indicating a synergism between the two ingredients [11]. Apart from these interactions at cellular level, PSO could also potentiate the FX effect by improving the carotenoid solubility thereby increasing its absorption rate and bioavailability to further enhance its anti-obesity effect [12,13].

As part of the registration process in Korea, a full assessment of the safety of Xanthigen^®^ was carried out using a standard battery of test for genotoxic activity (Ames assay, chromosomal aberrations assay using Chinese hamster ovary cells, and in vivo mammalian micronucleous test) and a rodent model for sub-chronic toxicity evaluation.

## 2 Materials and methods

### 2.1 Test product

The test product, Xanthigen^®^, was manufactured by Nektium Pharma S.L. (Canary Islands, Spain) and supplied by Rexgene Biotch Co., LTD (Ochang-eup, Cheongwon-gun, Chungbuk, Korea). It is sold as a dietary supplement for weight management. Xanthigen^®^ is a nutraceutical combination prepared from mixing equal amounts of brown seaweed extract (*Undaria pinitifida*) standardized to ≥0.85% fucoxanthin (to give a final concentration of 0.425% wt. in Xanthigen^®^) and pomegranate seed oil (*Punica granatum*) extract ≥ 70% punicic acid (35% wt. in final product). Xanthigen^®^ was manufactured in compliance with Good Manufacturing Practices (cGMPs) and retainer samples were kept for quality assurance purposes.

### 2.2 Bacterial reverse mutation (Ames) study

Xanthigen^®^ was evaluated using the *S. typhimurium* reverse mutation including four histidine-requiring strains of *S. typhimurium* (TA98, TA100, TA1535, TA1537) and *E. coli* reverse mutation assay with a tryptophan-requiring strain of *E. Coli* (WP2 uvrA). No more than 60 min prior to the start of the experiments, Xanthigen^®^ was weighed and dissolved in DMSO in a water bath (50 °C) and then agitated in a vortex mixer. The study was performed in two independent experiments both with and without metabolic activation (Aroclor 1254-induced rat liver S9-mix). A previous cytotoxicity evaluation was performed using 5–5000 μg/plate to determine the appropriate dose range for the assay. Doses up to 5000 μg/plate did not show antiproliferative activity against bacterial and therefore was the highest dose used for Ames assay.

Xanthigen was tested for its mutagenic potency using the plate incorporation method [14]. Briefly, after bacterial frozen stock cultures were grown overnight at 37 ± 2 °C until bacterial density reach 10^9^ cells/ml, sterile culture tubes were filled with 0.1 ml of either test Article (containing 5–5000 μg of Xanthigen^®^) or negative control, 0.5 ml of either S9/cofactor mix or phosphate buffer (pH 7.4), and 2.0 ml top agar. The mixture obtained was poured onto the surface of a plate containing minimal glucose agar medium and, after the top agar had hardened, incubated at 37 ± 2 °C for 72 ± 4 h. The number of revertant colonies per plate was counted in the treated groups, negative and positive controls with and without S9. The positive controls in the experiment without S9 activation were 2-nitrofluorene (CAS#607-57–8) for strain TA98, sodium-azide (CAS#26628-22-8) for strain TA100 and TA1535, 9-aminoacridine (CAS#52417-22-8) for strain TA1537, and methyl-methanesulfonate (CAS#66-27-3) for strain WP2. The positive controls for the experiment with S9 activation included benzo[a]pyrene (CAS#50-32-8) for strains TA98 and TA1537, and 2-aminoanthracene (CAS#613-13-8) for strains TA100, TA1535 and WP2. A negative control containing DMSO for measure basal spontaneous revertant colonies were included.

Criteria for a valid assay included the sensitivity of TA98, TA100, TA1535, TA1537 and WP2 to UV light, the sensitivity of *S. typhimurium* strains to crystal violet, the resistance of strains TA98 and TA100 to ampicillin, reversion rates within historical ranges, and a threefold increase in revertant colonies with exposure to positive controls. A response was considered positive if the number of revertant colonies on a plate was greater than three times the background average (twice background average for strain TA100), or if there was a dose-related increase in colonies. If these criteria were not met, the test product was considered non-mutagenic. Statistical regression analysis is not necessary in the absence of any test product related increase in the average number of revertant colonies relative to the negative control or of any dose-related increase.

### 2.3 In vitro chromosome aberration test

Chinese Hamster Ovary cells (CHO-k1) were obtained from Korean Cell Line Bank (KCLB) and test facility was performed by Bioconvergence technology laboratory, Korea Conformity Laboratories (Incheon, Korea). Cells used in the assay were within four passages from frozen stock to assure karyotypic stability. Cells were maintained in F-12 Nutrient Mixture (GIBCO, Thermo Fisher Scientific Inc., Waltham, Ma, USA) with 10% Fetal Bovine Serum (Hyclone, Logan, Utah, USA), 2 mM L-glutamine and 1% penicillin-streptomycin solution in a humidified incubator at 37 °C and with 5% CO_2_ enriched air. The post-mitochondrial fraction (S9) of the liver homogenate from Sprague-Dawley rats induced with Arclor 1254 (Monsanto Co., St. Louis, MO, USA) was used for metabolic activation at final concentration of approximately 1% by vol/vol. The compound 1,4 dioxane was chosen as solvent because its compatibility with the test system and Xanthigen^®^ found to be soluble at maximum dose level (5000 μg/mL).

For chromosome aberration assay, 48 h after seeding, CHO-k1 cells were treated with Xhantigen^®^ dissolved to a concentration of 625, 1250 and 2500 μg/mL in F-12. These concentrations were selected on the basis of preliminary cytotoxicity study where doses of the test article that produce value higher than 20% of cytotoxicity were discarded.

Three treatment schemes were used, including incubation of test articles without S9 metabolic activation system for 24 h (Scheme I) and incubation for 6 h with 18 h of recovery both in the presence or absence of S9 metabolic activation system (Scheme II and III, respectively). Positive controls prepared in DMSO, selected according to OECD guidelines, were included: Mitomycin (MMC) at 0.04 μg/μL (without metabolic activation) and cytophosphadine*H_2_O (CPA) at 10 μg/μL (with metabolic activation). As negative control, 1.4 dioxane was used since this was the solvent used to dissolve Xanthigen^®^.

Colcemid (0.2 μg/mL) was added to all cell culture wells 2 h before harvesting in order to induce the cell division arrest. Thereafter, the cells were evaluated for chromosomal integrity by cell counting on previous fixed and staining slides that were blind coded. Two cultures were scored for each dose and 100 metaphases were observed for each culture. Chromosomal and chromatid aberrations were scored, and the percentage of structurally aberrant cells was calculated, excluding cells with chromosome or chromatid gaps.

If the number of aberrant cells observed was greater than 3%, the result was interpreted with a one-tailed binomial test. A Pearson´s chi-squared test was applied to the negative control and treated groups, with a separate chi-squared test carried out on the negative and positive control groups. A linear logistic regression test was performed for dose versus response, with a positive response considered to be a dose-related and statistically significant increase in the number of aberrant metaphases, or where reproducible positive results were detected in at least one test concentration. The statistical analyses were performed with the SPSS 12.1 K software package at the 5% significance level.

### 2.4 Micronucleus assay in mice

Xanthigen^®^ was evaluated for its ability to induce micronucleus in the polychromatic erythrocytes (PCE) of the bone marrow from mice following oral administration of the test product (OECD test No. 474, 2014). Male SPF (specific pathogenic free) ICR mice (CD-1), with an age of 7–8 weeks, were supplied by Orient Bio Co., Ltd. (Gyeonggi, Korea). Body weights at the start of the study were in the range 33.15 ± 1.13 g–33.79 ± 0.89 g. The animals were randomly using their graded body weight, assigned to five groups of six, housed in cages with three animals in each and checked for signs of illness and other abnormalities prior to the start of the study. Conditions were maintained at 56.1 ± 1.8% relative humidity, 21.6 ± 0.8 ℃ and with a 12-h light-dark cycle. Animals had *ad libitum* access to drinking water and, via a feeding supplier, to a radiation sterilized and pelleted diet (DooYeol Biotech Co., Korea). Body weight was measured on the date of Xanthigen administration and at autopsy.

The test product was dissolved in a vehicle of corn oil for oral administration by oral gavage, with a dosage of 10 ml/kg based on the body weight measured on the same day. A preliminary dose-range finding study was conducted to determine the proper dosage and sampling time for the main study. Mouse were treated with 500, 1000 and 2000 mg/kg of Xanthigen or corn oil (negative control) for 24 or 48 h. The positive control, 2 mg/kg of Mitomycin C (MMC), was administered intraperitoneally (i.p.) to the test animals using a disposable 26 G syringe. As no sign of acute toxicity or bone marrow cytotoxicity were observed, doses up to 2000 mg/kg were selected for the main assay.

In order to perform the micronucleus assay, Xanthigen^®^ was dissolved in corn oil to get a final concentration of 5, 10 and 20% w/v. Mouse were oral gavage with a Xanthigen^®^ suspension at a volume of 10 ml/kg (Xanthigen doses of 500, 1000 and 2000 mg/kg). Animals receiving the same amount of vehicle were included as negative controls. MMC used as positive control was dissolved and administered i.p. Harvesting of bone marrow was conducted 24 h after oral administration and smeared onto a glass slide. Three slides were prepared for each animal. The thigh bone of the test animal was extracted with care to avoid contamination with blood in the autopsy room after removal of the cervical vertebra. To avoid bias, observation of the slides was performed blinded. To score the PCEs with micronuclei (MNPCEs) from PCEs, the slides were fixed and stained with acridine orange (40 μg/ml). An optical microscope at 400× magnification (200 erythrocytes per animal) was used. MNPCEs were observed with a fluorescence microscope equipped with a FITC filter. The micronuclei frequency (the percentage of micronucleated cells) was determined by analysing the number of MNPCEs from 2000 PCEs per animal. To check potential cytotoxic effects, the ratio of polychromatic erythrocytes (PCEs) to normochromatic erythrocytes (NCEs) in 2000 erythrocytes was calculated.

Statistical analysis was performed with the SPSS 12.0 software package and *p* values of less than 0.05 were considered to indicate significant results. With micronuclei frequency (MNPCE / 2000PCEs, mean ± SD, %) and PCE / (PCE + NCE) ratio (mean ± SD), the groups were compared using one-way analysis of variance (ANOVA). In cases with p < 0.05, a linear logistic regression was used to test for dose-response. ANOVA was used to compare body weight among groups. In case of significant differences, Dunnett T3 or Duncan's multiple range tests were applied.

### 2.5 14-days acute oral toxicity study in rats

The acute toxicity of Xanthigen was studied in 6-week old Specific Pathogen Free (SPF) Sprague-Dawley (SD) rats obtained from ORIENT BIO INC. (Gyeonggi-do, Korea). Rats were housed three per cage at a temperature of 22 ± 7 °C, a relative humidity of 53.4 ± 5.2%, and subjected to a 12 h light–dark cycle. Animals received radiation sterilized, solid laboratory animal feed for rats, produced by Harlan Laboratories, Inc., (USA) and tap water, both of which were available *ad libitum*. Randomization and statistical analysis was performed with SPSS v.12.0 (SPSS Inc., Chicago, IL, USA). The differences between the vehicle control and the dosing groups were examined using parametric multiple comparison procedures or non-parametric multiple comparison procedures and the occurrence rate was converted into a percentage.

Body weights at randomization were 206.54–236.86 g for males, and 140.63–177.80 g for females. Each group consisted of 5 males and 5 females. Detailed clinical observations were made prior to exposure of the animals to the test article. On the day of administration, the Xanthigen^®^ was dissolved in corn oil to give a high dose test solution (2000 mg/kg) and then two other doses of 1000 and 500 mg/kg were prepared by serial dilution. For each concentration, a single oral dose was administered to 6-weeks old rats by gavage every morning for 2 weeks. The general clinical signs were noted for all treated animals daily after feeding. Individual records were maintained for each animal that included food consumption, ophthalmoscopy, urinalysis, haematology, blood biochemistry, mortality, type, date and grade of clinical signs and necropsy. The individual’s weight was recorded at acquisition, grouping, before administration, once a week during the study and before necropsy. At necropsy, the weights of below organs were recorded. This study was performed in compliance with the Korea Food and Drug Administration (KFDA) Notification No. 2009-116′Toxicity Test Standards of Medicine and Medical Supplies' (Revised 24th August 2009) and OECD Guideline for the Testing of Chemicals No. 407′Repeated Dose 28-day Oral Toxicity Study in Rodents' (Adopted 27th July 1995).

### 2.6 Subchronic 90-day oral toxicity study in rats

SPF SD rats with an age of 6 weeks were obtained from ORIENT BIO INC. (Gyeonggi-do, Korea). The animals were housed one or two per cage, maintained at a temperature of 21.6 ± 0.9 °C and 57.4 ± 3.6% relative humidity, and subjected to a 12-h light–dark cycle. Animals received radiation sterilized solid laboratory animal feed for rats, produced by Harlan Laboratories, Inc., (USA) and both food and tap water were accessible *ad libitum*. The body weights of the animals at randomization were 178.77–217.82 g for males, and 140.26–166.90 g for females. An equal number of animals from each sex and weight group were randomly assigned to each of the two experimental groups. Each group (G1, G2, G3 and G4) consisted of 10 males and 10 females. Doses of 0 (G1), 250 (G2), 500 (G3) and 1000 (G4) mg/kg/day of Xanthigen^®^ in a volume of 5 ml/kg was force fed every morning for 90 days. Weekly adjustments were made for changes in body weight. Every day of administration, the test article was weighed accurately and dissolved in corn oil (vehicle) to formulate the high dose (1000 mg/kg) test solution that was then serially diluted to produce the 500 and 250 mg/kg doses.

Daily clinical observations were made following each treatment at approximately the same time each day. Individual records were maintained for each animal including the mortality, type, date and grade of clinical signs. Observations are described in the acute oral toxicity study in Section 2.5. All animals were weighed on the day of randomization, on the first day of the study, weekly during the study, and immediately before necropsy. Food was weighed weekly, and the average food consumption per animal calculated. Haematology and clinical chemistry results were evaluated upon termination of the study. Prior to necropsy, the animals were fasted overnight (16 h) and then blood samples were drawn from the abdominal aorta, collected in tubes and centrifuged to obtain the serum. Once animals were sacrificed with CO_2_, a gross pathological examination was performed on all organs after death, including organ weight and appearance. A histopathological examination was conducted on the control group and the highest dose group, and on the middle dose groups when appropriate. The serum biochemistry parameters (Table 4) were analysed with a multi-channel automatic analyser Hitachi 7180 (Hitachi Ltd., Tokyo, Japan) for lipids/lipoproteins, enzymes and metabolites (Table 4) or with the electrolyte analyzer Roche AVL9181 for electrolyte determinations using commercially available Roche test kits (Roche Diagnostic, Basel, Switzerland).

Statistical analysis was performed by SPSS v.12 for window (SPSS Inc., Chicago, IL USA). The differences between the vehicle control and dosing groups were examined using parametric or non-parametric multiple comparison procedures. The occurrence rate was converted into a percentage. Analysis of continuous variables (body weight, food consumption, haematology, blood coagulation time, blood biochemistry, organ weight) was performed by one-way ANOVA followed by multiple comparison (Duncan’s test if the variance were considered homogeneous or Dunnett´s test if variance was unequal). For the analysis of categorical variables (urinalysis), a chi-squared test was applied after rescaling.

The study was performed in accordance with Korea Food and Drug Administration (KFDA) “Principles of Good Laboratory Practice” (No. 2009-183); “Toxicity and Test Standard of Medicine and Medical Supplies” (No. 2009-116), as well as the OECD Guidelines for the Testing of Chemicals No. 408; Repeated Dose 90-Day Oral Toxicity Study in Rodents, adopted September 21, 1998.

## 3 Results

### 3.1 Bacterial reverse mutation (Ames) study

The bacterial reverse mutation assay was performed to evaluate whether Xanthigen^®^ has mutagenic properties. No cytotoxic effect was observed either with or without 4% S9 activator at any of the five selected doses of Xanthigen^®^ (data not shown) and in any case did revertants exceed three times the background average in either with or without the S9 metabolic activation system (Table 1). In addition, no dose-dependent increase in revertants was observed. In conclusion, the results of the Ames test showed that Xanthigen^®^ had no mutagenic effect for any strain used in this test. Furthermore, the results of the repeated assay confirmed the results of the definitive assay.

**Table 1:** Ames test of Xanthigen using Salmonella Typhimurium (TA98, TA100, TA1535, TA1537) and Escherichia Coli (WP2) strains.

Doses (μg/plate)	Number of revertants/plate (*Without S9 activation*)				
	TA98	TA100	TA1535	TA1537	WP2(uvrA)
50	31 ± 3.0	178 ± 6.7	30 ± 3.5	14 ± 2.6	74 ± 4.0
100	32 ± 4.7	178 ± 4.5	28 ± 4.6	12 ± 3.5	72 ± 7.9
500	32 ± 3.5	184 ± 4.0	30 ± 1.5	15 ± 1.5	73 ± 4.5
1000	33 ± 6.7	179 ± 7.8	28 ± 2.5	13 ± 2.0	76 ± 4.0
5000	31 ± 4.0	178 ± 10.1	29 ± 2.6	15 ± 3.5	73 ± 4.0
Negative control^a^	33 ± 2.5	180 ± 6.7	30 ± 6.4	14 ± 4.6	74 ± 4.9
Positive control^b^	465 ± 29.5*	896 ± 66.8*	431 ± 29.7*	64 ± 5.3*	1041 ± 43.6*
	A	B	B	C	D
	Number of revertants/plate (*With S9 activation)*				
	TA98	TA100	TA1535	TA1537	WP2 (uvrA)
50	48 ± 7.2	161 ± 10.0	31 ± 6.1	11 ± 2.5	78 ± 4.6
100	52 ± 3.1	165 ± 8.3	32 ± 8.7	10 ± 1.0	80 ± 7.0
500	50 ± 8.5	165 ± 10.6	31 ± 2.6	11 ± 1.0	79 ± 5.6
1000	47 ± 7.6	163 ± 5.3	31 ± 4.0	11 ± 3.5	80 ± 7.8
5000	50 ± 3.5	160 ± 11.4	28 ± 4.2	10 ± 1.0	79 ± 6.5
Negative control^a^	51 ± 4.5	162 ± 10.7	32 ± 4.6	10 ± 1.5	80 ± 5.0
Positive control^b^	208 ± 19.0*	501 ± 44.8*	389 ± 21.4*	138 ± 7.0*	451 ± 51.0
	E	F	F	E	F

Data represent the means and standard deviation of the results obtained from three independent experiments.

* Three-fold or more increase in revertant numbers over the negative control.

a Dimethyl sulfoxide.

b Types and concentrations of positive control groups used in different strains: A, 2-nitrofluorene (2.5 μg/plate); B, sodium- azide (1.5 μg/plate); C, 9-aminoacridine (25 μg/plate); D, methyl-methansulfonate (2.5 μg/plate); E, benzo[*a*]pyrene (20 μg/plate); F, 2-aminoanthracene (10 μg/plate).

### 3.2 In vitro chromosome aberration test

The chromosome aberration assay in cultured mammalian cells (CHO-k1) was performed to check whether the test article causes structural damage to either chromosomes or chromatids. The results of this assay are summarized in Table 2. In both the presence and absence of S9 metabolic activation and at all of the Xanthigen^®^ dose levels tested, no statistically significant increase in the number of cells with chromosome aberration relative to the negative controls was observed, while in the positive controls, the expected increase relative to the negative controls was detected. Therefore, under the conditions used in this test, Xanthigen^®^ did not induce structural chromosomal aberrations either with or without the metabolic activation system in CHO-k1 cells.

**Table 2:** Chromosome aberration test of Xanthigen using CHO-K1 cells.

Concentrations (μg/mL)	Chromosomal aberration (%)		
	With S9, 6h Treatment^d^	Without S9, 6 h treatment^d^	Without S9, 24 h treatment
Negative control^a^	0.5	0.5	0.5
Positive control^b^, ^c^	25.5*	20*	24*
2500	0.5	1	0.5
1250	1	0.5	1.5
625	0	0.5	1.5

a Culture medium containing 10% fetal bovine serum.

b 6 μM mitomicyn C was used without S9.

c 80 μM cyclophosphamide monohydrate was used with S9.

d Plus 18 h of recovery time.

* Statistically significant when compared to the negative control group, *p* <0.05.

### 3.3 Micronucleus assay in mice

The micronucleus test was conducted to investigate the formation of micronuclei-containing chromosome fragments or whole chromosomes, which are indicative of cytogenetic damage. A summary of results from micronucleus assay is shown in Table 3. No differences in body weight were measured between the treatment groups and the negative control group and no clinical signs of toxicity were observed following administration of any test product dose. The frequencies of MNPCEs in 2000 PCEs per animal were 0.16%, 0.15%, 0.25%, 0.20% and 10.87% for the negative control group, 500, 1000, 2000 mg/kg administration group and positive control group, respectively. There was no statistically significant increase in frequency of PCEs with micronuclei in the administration group relative to the negative control group, while there was a clear and statistically significant increase in the positive control group compared with the negative control group in frequency of MNPCE (p < 0.01). The PCE ratio of 200 erythrocytes (PCE + NCE), as an index of cytotoxicity, was 0.45, 0.45, 0.50, 0.49 and 0.38 for the negative control group, the 500, 1000, 2000 mg/kg group and positive control group, respectively. There was no significant decrease in PCE/(PCE + NCE) ratio in the Xanthigen^®^ treated group compared to the negative control group.

**Table 3:** In vivo mouse micronucleus and weights data summary table using Xanthigen, data are showed as mean ± standard deviation.

Body weights (gram, mean ± SD)				
Dose (mg/kg)	% Micronucleated PCEs (mean^b^ ± SD)	Ratio PCE:NCE (mean ± SD)	Administration	Sacrifice
0 (Vehicle control^a^)	0.16 ± 0.06	0.45 ± 0.04	33.74 ± 0.89	33.87 ± 0.96
500	0.15 ± 0.13	0.45 ± 0.09	33.53 ± 1.29	33.82 ± 1.26
1000	0.25 ± 0.07	0.50 ± 0.04	33.32 ± 1.27	33.58 ± 1.08
2000	0.20 ± 0.03	0.49 ± 0.04	33.58 ± 1.17	33.79 ± 1.12
2.0 (positive control^c^)	10.87 ± 1.08*	0.38 ± 0.03*	33.15 ± 1.13	33.51 ± 1.19

a Corn oil.

b Mean of 2000 micronucleated PCEs per mouse.

c Mitomycin C.

* Statistically different from vehicle control group, *p*<0.01.

### 3.4 14-day oral toxicity study in rats

A 14-day chronic toxicity study in rats was performed with Xanthigen^®^ in order to provide preliminary toxicity data that are useful for determining appropriate dosages for future repeated-dose oral toxicity studies as well as for identifying organs that should be closely monitored in such studies. Oral doses of 2000, 1000 and 500 mg/kg/day for 2 weeks induced no treatment-related signs in any of the animals. No changes in body weight were observed in the treatment group compared with the control group in both males and females (Table 4). No abnormal lesions were observed in any animals during necropsy. In the urine analysis, the only significant differences (*p* < 0.05) were observed for specific gravity and pH in female rats. In the blood biochemistry of the males, an increase in ALT value was observed but this did not show a dose-dependent relationship (63 ± 6.3IU/L in low dose vs control (48 ± 5.2 IU/L), 1000 mg/kg (49 ± 5.5 IU/L) and 2000 mg/kg (53 ± 9.5IU/L); group; *p* < 0.05). Total cholesterol in the high dose male group (105 ± 21.7 mg/dl) was significantly higher (*p* < 0.05) than in controls, 500 mg/kg and 1000 mg/kg group (78 ± 18.1 mg/dL, 80 ± 14.1 md/dL and 99 ± 7.3 mg/dL, respectively; p < 0.05). A similar result was observed among female rats and tended to increase with increasing dose (72 ± 11.9 mg/dl in the control group, 90 ± 21 mg/dl in the 500 mg/kg group, 114 ± 19.6 mg/dl in 1000 mg/kg and 138 ± 22.4 mg/dl in 2000 mg/kg, p < 0.001). In the female group, the TG values (*p* < 0.05) increased and tended to be higher than those of the control group at 500 and 1000 mg/kg doses (19 ± 4.2 mg/dL, 30 ± 12.4 mg/dL, 37 ± 13.8 mg/dl and 48 ± 11.2 mg/dl from 0 to 2000 mg/kg groups). Therefore, these changes were not considered to be toxicologically significant and no other significant correlations were observed.

**Table 4:** Summary of mean organ weight (g) and organ weight relative to body weight (%) for male and female rats in the 2 weeks oral repeated dose finding study.

		Control (G1)	500 mg/kg/day (G2)	1000 mg/kg/day (G3)	2000 mg/kg/day (G4)
*Males*					
Body	g	313.21 ± 25.82	317.80 ± 17.01	341 ± 14.96	318 ± 20.10
Testis (Lt.)	g	1.40 ± 0.09	1.46 ± 0.15	1.48 ± 0.06	1.51 ± 0.06
	%	0.45 ± 0.05	0.46 ± 0.03	0.43 ± 0.0	0.47 ± 0.03
Testis (Rt.)	g	1.41 ± 0.06	1.49 ± 0.13	1.49 ± 0.10	1.53 ± 0.06
	%	0.45 ± 0.04	0.46 ± 0.02	0.43 ± 0.04	0.48 ± 0.02
Prostate	g	0.52 ± 0.20	0.42 ± 0.06	0.44 ± 0.07	0.51 ± 0.14
	%	0.16 ± 0.07	0.13 ± 0.001	0.13 ± 0.02	0.16 ± 0.04
Spleen	g	0.76 ± 0.09	0.75 ± 0.07	0.77 ± 0.05	0.72 ± 0.08
	%	0.24 ± 0.04	0.23 ± 0.02	0.22 ± 0.02	0.22 ± 0.03
Liver	g	11.01 ± 2.06	11.72 ± 1.50	12.54 ± 0.49	11.69 ± 1.26
	%	3.49 ± 0.40	3.68 ± 0.33	3.67 ± 0.23	3.66 ± 0.19
Adrenal (Lt.)	g	0.026 ± 0.004	0.023 ± 0.006	0.025 ± 0.002	0.023 ± 0.005
	%	0.008 ± 0.001	0.007 ± 0.001	0.007 ± 0.002	0.007 ± 0.001
Adrenal (Rt.)	g	0.024 ± 0.003	0.022 ± 0.002	0.024 ± 0.002	0.022 ± 0.005
	%	0.007 ± 0.001	0.007 ± 0.000	0.007 ± 0.000	0.007 ± 0.002
Kidney (Lt.)	g	1.27 ± 0.12	1.27 ± 0.09	1.33 ± 0.04	1.18 ± 0.14
	%	0.40 ± 0.03	0.40 ± 0.02	0.39 ± 0.01	0.37 ± 0.07
Kidney (Rt.)	g	1.33 ± 0.09	1.33 ± 0.10	1.41 ± 0.05	1.18 ± 0.10ª
	%	0.42 ± 0.03	0.41 ± 0.0)	0.41 ± 0.01	0.37 ± 0.02^a^
Heart	g	1.20 ± 0.15	1.21 ± 0.15	1.98 ± 0.10	1.26 ± 0.07
	%	0.38 ± 0.05	0.38 ± 0.03	0.35 ± 0.03	0.40 ± 0.05
Lung	g	1.88 ± 0.44	1.65 ± 0.24	1.77 ± 0.31	1.84 ± 0.32
	%	0.60 ± 0.13	0.52 ± 0.08	0.52 ± 0.05	0.57 ± 0.08
Brain	g	2.03 ± 0.08	2.06 ± 0.05	1.98 ± 0.10	1.92 ± 0.06
	%	0.65 ± 0.04	0.65 ± 0.03	0.58 ± 0.05^b^	0.60 ± 0.0
Thymus	g	0.54 ± 0.14	0.65 ± 0.14	0.61 ± 0.10	0.55 ± 0.13
	%	0.17 ± 0.03	0.20 ± 0.03	0.17 ± 0.03	0.17 ± 0.03
		Control	500 mg/kg/day	1000 mg/kg/day	2000 mg/kg/day
*Females*					
Body	g	204.33 ± 25.74	204 ± 20.20	203.24 ± 7.29	199.28 ± 3.91
Ovary (Lt.)	g	0.03 ± 0.006	0.04 ± 0.009	0.033 ± 0.008	0.030 ± 0.006
	%	0.014 ± 0.003	0.019 ± 0.004	0.016 ± 0.004	0.015 ± 0.003
Ovary (Rt.)	g	0.04 ± 0.009	0.04 ± 0.018	0.030 ± 0.004	0.033 ± 0.004
	%	0.014 ± 0.004	0.02 ± 0.008	0.015 ± 0.002	0.016 ± 0.002
Uterus	g	0.36 ± 0.04	0.58 ± 0.20	0.33 ± 0.03c	0.42 ± 0.04
	%	0.18 ± 0.01	0.29 ± 0.11	0.16 ± 0.01^c^	0.21 ± 0.02
Spleen	g	0.50 ± 0.09	0.52 ± 0.06	0.52 ± 0.09	0.45 ± 0.12
	%	0.24 ± 0.01	0.25 ± 0.009	0.25 ± 0.04	0.22 ± 0.05
Liver	g	7.34 ± 1.37	7.42 ± 1.00	7.05 ± 0.59	7.52 ± 0.11
	%	3.57 ± 0.24	3.61 ± 0.16	3.48 ± 0.21	3.77 ± 0.08
Adrenal (Lt.)	g	0.02 ± 0.006	0.03 ± 0.004	0.029 ± 0.008	0.028 ± 0.005
	%	0.014 ± 0.002	0.014 ± 0.001	0.014 ± 0.004	0.014 ± 0.002
Adrenal (Rt.)	g	0.02 ± 0.005	0.028 ± 0.003	0.028 ± 0.005	0.024 ± 0.006
	%	0.014 ± 0.001	0.013 ± 0.000	0.014 ± 0.003	0.012 ± 0.003
Kidney (Lt.)	g	0.82 ± 0.11	0.82 ± 0.12	0.83 ± 0.09	0.82 ± 0.03
	%	0.40 ± 0.02	0.40 ± 0.04	0.41 ± 0.04	0.41 ± 0.01
Kidney (Rt.)	g	0.82 ± 0.09	0.82 ± 0.10	0.87 ± 0.10	0.83 ± 0.08
	%	0.40 ± 0.03	0.40 ± 0.03	0.43 ± 0.02	0.41 ± 0.03
Heart	g	0.76 ± 0.08	0.84 ± 0.09	0.85 ± 0.04	0.76 ± 0.03
	%	0.37 ± 0.02	0.41 ± 0.02^d^	0.42 ± 0.02^c^, ^d^	0.38 ± 0.01
Lung	g	1.27 ± 0.12	1.33 ± 0.34	1.18 ± 0.07	1.48 ± 0.09
	%	0.63 ± 0.09	0.64 ± 0.11	0.58 ± 0.03	0.74 ± 0.06
Brain	g	1.80 ± 0.08	1.77 ± 0.09	1.79 ± 0.09	1.80 ± 0.07
	%	0.89 ± 0.09	0.87 ± 0.07	0.88 ± 0.04	0.90 ± 0.02
Thymus	g	0.54 ± 0.16	0.57 ± 0.09	0.60 ± 0.14	0.47 ± 0.072
	%	0.26 ± 0.06	0.28 ± 0.02	0.30 ± 0.07	0.23 ± 0.03

abbreviationd: Rt = Right, Lt = Left.

Values are means ± SD.

a Significant difference compared with control, 500, 1000 mg/kg group value, p < 0.05.

b Significant difference compared with control, 500 mg/kg group value, p < 0.05.

c Significant difference compared with 2000 mg/kg group value, p < 0.05.

d Significant difference compared with control group value, p < 0.05.

With respect to the weights of organs, the right kidneys (absolute and relative, *p* < 0.05) and brains (relative, *p* < 0.05) in males were significantly lighter than those of the control group. Among the G2 and G3 female groups, heart weight (relative, *p* < 0.05) was greater than in the other two groups. Uterus weight in G3 (absolute and relative, p < 0.05) was significantly less than that of the high dose group and brain weights in the G2 and G3 groups were heavier than in the other groups (relative, p < 0.05). But these changes were not considered to be a response to the test substance since no dose-dependent relationship was apparent (Table 4). No other effects related to the test substance were observed in any of the other organs that were inspected.

In summary, the test substance, Xanthigen^®^, exhibited no toxic effects during the 14-day repeated oral dose toxicity study. Consequently, a general maximum dose of 1000 mg/kg was selected for the 90-day oral repeated dose toxicity study.

### 3.5 Subchronic 90-day oral toxicity study in rats

A 90-day repeated-dose toxicity study was performed in rats to determine a NOAEL for defined toxicological endpoints and to establish a safe chronic oral dose for humans. Xanthigen^®^ was administered by oral gavage at doses of 250, 500 and 1000 mg/kg/day, for 90 consecutive days (13 weeks). Result are shown in Table 5, Table 6, Table 7, Table 8, Table 9. No deaths or treatment-related effects were observed throughout the 90-day treatment period in any of the groups. Significant decreases in body weight were observed in males in groups G2 (at 5, 8, 10, 11 and 12 weeks; p < 0.05) and G3 (at 8 weeks; p < 0.05) and in females in groups G2 (at 8, 10, 11 and 13 weeks; p < 0.05, at 9 and 12 weeks and at necropsy, p < 0.01) and G4 (from 4 to 13 weeks; p < 0.05, and at necropsy; p < 0.01) throughout the study period (Table 2). However, it was considered that these changes were caused by the effect of Xanthigen^®^ on weight loss, demonstrated in earlier studies. A significant (p < 0.05), albeit temporary, decrease in food consumption, at 8 weeks in males in groups G2 (26.93 ± 2.11 g) and G3 (28.14 ± 2.53 g) vs the control (32.32 ± 4.34 g) and in females in group G4 (15.01 ± 1.65 g) vs the control group (20.32 ± 3.19 g) was also observed (Table 6). Nevertheless, these changes were not considered to be related to the test.

**Table 5:** Summary of Body weight (BW) and Fold weight gain (FWG) data in rats’ subchronic 13-week oral toxicity study.

Weeks		Control(G1)	250 mg/kg/day(G2)	500 mg/kg/day (G3)	1000 mg/kg/day (G4)
*Males*					
0	BW	202.46 ± 9.45	201.84 ± 10.77	202.42 ± 9.11	202.77 ± 4.50
1	BW	248.70 ± 13.66	235.57 ± 16.43	245.00 ± 9.61	249.68 ± 6.39
2	BW	312.45 ± 18.15	299.83 ± 20.12	303.30 ± 12.59	312.65 ± 14.14
3	BW	372.53 ± 26.85	347.42 ± 26.67	353.10 ± 27.26	372.04 ± 20.08
4	BW	422.97 ± 34.18	388.29 ± 31.67	401.36 ± 32.91	420.33 ± 26.58
5	BW	468.66 ± 40.56	422.46 ± 36.92^a^	437.80 ± 36.20	461.07 ± 30.97
6	BW	503.00 ± 42.97	458.75 ± 38.40	466.91 ± 42.72	493.94 ± 34.92
7	BW	525.85 ± 40.36	487.86 ± 44.87	494.97 ± 43.86	521.89 ± 35.98
8	BW	562.85 ± 48.95	509.94 ± 46.15^a^	519.03 ± 46.44^a^	551.59 ± 38.98
9	BW	581.21 ± 53.69	532.69 ± 49.30	539.71 ± 49.38	575.74 ± 45.72
10	BW	603.21 ± 54.32	543.32 ± 51.94^a^	559.88 ± 51.39	596.04 ± 49.50
11	BW	625.75 ± 55.99	567.04 ± 51.94^a^	577.27 ± 54.26	616.77 ± 50.35
12	BW	644.76 ± 61.49	583.34 ± 54.09^a^	591.18 ± 53.33	639.42 ± 56.59
13	BW	654.38 ± 66.20	594.59 ± 54.67	609.99 ± 56.82	650.64 ± 61.70
necropsy	BW	640.31 ± 62.52	582.92 ± 53.51	595.07 ± 56.88	631.75 ± 63.42
	FWG	3.16	2.89	2.94	3.12

*Females*					
0	BW	155.33 ± 9.10	156.95 ± 5.62	155.38 ± 7.35	156.07 ± 7.46
1	BW	180.64 ± 12.00	177.75 ± 12.02	177.19 ± 8.40	173.43 ± 10.64
2	BW	207.73 ± 14.35	202.38 ± 15.52	195.78 ± 9.80	192.05 ± 14.91
3	BW	226.91 ± 19.24	219.40 ± 21.69	223.27 ± 14.99	210.75 ± 16.43
4	BW	251.02 ± 23.11	239.00 ± 25.26	245.71 ± 16.03	225.50 ± 16.09^a^
5	BW	269.60 ± 23.02	255.92 ± 30.13	260.43 ± 13.91	241.28 ± 20.91
6	BW	283.77 ± 21.33	267.19 ± 27.14	271.16 ± 13.64	253.77 ± 20.9^a^
7	BW	291.93 ± 22.95	274.01 ± 25.13	284.89 ± 14.66	261.00 ± 23.81^a^
8	BW	305.96 ± 28.27	281.20 ± 29.85^a^	291.56 ± 15.55	269.96 ± 20.30^a^
9	BW	314.89 ± 26.89	287.51 ± 29.49^b^	302.35 ± 15.81	277.51 ± 24.02^b^
10	BW	321.04 ± 26.41	294.37 ± 29.21^a^	305.54 ± 14.37	284.36 ± 24.20^a^
11	BW	328.95 ± 27.72	298.70 ± 35.45^a^	312.87 ± 13.69	288.90 ± 24.92^a^
12	BW	334.37 ± 31.63	302.94 ± 35.32^b^	321.13 ± 13.57	288.67 ± 22.55^b^
13	BW	338.62 ± 28.76	308.19 ± 35.50^a^	324.79 ± 13.44	297.94 ± 25.91^a^
necropsy		325.21 ± 25.90	295.89 ± 34.91^b^	313.71 ± 14.59	286.54 ± 24.63^b^
	FWG	2.09	1.89	2.02	1.84

Values are Mean ± SD.

a Significant difference compare with control group value, p < 0.05.

b Significant difference compare with control value, p < 0.01.

**Table 6:** Summary of food consumption data in rats’ subchronic 13-week oral toxicity study.

Weeks	Control (G1)	250 mg/kg/day (G2)	500 mg/kg/day (G3)	1000 mg/kg/day (G4)
*Males*				
0	24.25 ± 1.83	25.00 ± 1.82	20.75 ± 7.80	25.26 ± 1.85
1	27.90 ± 2.00	23.12 ± 6.65	26.24 ± 2.49	28.36 ± 1.26
2	29.92 ± 2.57	27.67 ± 1.56	28.41 ± 2.72	30.51 ± 3.15
3	31.48 ± 4.40	27.95 ± 2.42	29.33 ± 4.07	32.17 ± 3.43
4	33.44 ± 5.80	28.22 ± 2.68	30.92 ± 5.00	35.17 ± 8.09
5	31.88 ± 4.78	27.58 ± 2.58	28.69 ± 3.63	30.35 ± 2.56
6	30.43 ± 2.73	27.67 ± 1.52	27.42 ± 2.73	29.79 ± 2.04
7	29.33 ± 8.61	28.82 ± 1.17	29.12 ± 2.57	31.21 ± 2.39
8	32.32 ± 4.34	26.93 ± 2.11^a^	28.14 ± 2.53^a^	31.10 ± 2.16
9	28.29 ± 6.29	28.42 ± 3.29	27.74 ± 3.51	30.30 ± 2.92
10	28.42 ± 2.88	24.86 ± 5.63	27.11 ± 2.25	30.28 ± 1.19
11	28.88 ± 2.66	25.72 ± 1.69	26.48 ± 2.17	28.52 ± 1.83
12	28.36 ± 3.56	26.07 ± 2.23	25.00 ± 3.14	29.32 ± 2.16
13	25.31 ± 4.19	26.27 ± 2.21	25.20 ± 1.90	28.03 ± 1.31
Fold gain	1.04	1.05	1.21	1.11

Weeks	Control	250 mg/kg/day	500 mg/kg/day	1000 mg/kg/day
*Females*				
0	18.09 ± 2.02	20.19 ± 1.50	18.40 ± 1.73	18.76 ± 2.15
1	19.46 ± 3.24	18.17 ± 3.30	19.63 ± 2.06	17.44 ± 2.47
2	21.78 ± 3.01	20.06 ± 3.32	16.74 ± 3.32	17.53 ± 3.55
3	21.00 ± 1.20	19.61 ± 4.00	22.12 ± 2.68	19.86 ± 3.41
4	22.93 ± 3.55	20.35 ± 2.99	23.23 ± 2.24	18.84 ± 1.92
5	23.80 ± 2.24	20.96 ± 3.74	22.62 ± 2.16	21.76 ± 2.73
6	23.22 ± 2.00	21.16 ± 1.15	20.27 ± 4.37	19.91 ± 3.07
7	20.03 ± 2.86	17.78 ± 1.90	20.17 ± 1.84	18.13 ± 2.65
8	21.19 ± 4.37	17.25 ± 3.08	18.65 ± 3.17	17.69 ± 1.48
9	22.06 ± 2.39	18.03 ± 2.52	19.66 ± 2.11	19.23 ± 3.34
10	17.72 ± 2.00	16.21 ± 0.96	15.90 ± 3.46	17.47 ± 1.54
11	17.63 ± 1.31	16.87 ± 3.14	17.73 ± 3.71	16.79 ± 1.61
12	20.32 ± 3.19	17.66 ± 2.98	19.14 ± 1.59	15.01 ± 1.65^a^
13	18.03 ± 2.05	18.05 ±4.23	16.34 ± 2.26	16.75 ± 2.72
Fold gain	1.00	0.89	0.89	0.89

Values are Mean ± SD.

a Significant difference compare with control group value, p < 0.05.

**Table 7:** Summary of serum biochemical data in the rat subchronic 13-week oral toxicity study.

*Males*	AST (UI/L)	ALT (IU/L)	ALP (IU/L)	GGT (IU/L)	LDH(IU/L)
Control	110 ± 29	36 ± 8	270 ± 92	0.8 ± 0.4	1069 ± 679
250 mg/kg/day	138 ± 43	38 ± 9	279 ± 74	0.8 ± 0.4	1556 ± 909
500 mg/kg/day	120 ± 47	46 ± 27	291 ± 93	0.4 ± 0.5	1027 ± 911
1000 mg/kg/day	118 ± 35	33 ± 4	256 ± 60	0.8 ± 0.4	1367 ± 873

*Males*	**BUN (mg/dl)**	**CRE (mg/dl)**	**GLU (mg/dl)**	**CPK (U/L)**	**UA (mg/dl)**
Control	13.8 ± 1.5	0.56 ± 0.03	195 ± 18	501 ± 275	2.4 ± 0.7
250 mg/kg/day	13.6 ± 1.9	0.56 ± 0.04	192 ± 21	675 ± 321	2.3 ± 0.6
500 mg/kg/day	13.8 ± 2.0	0.54 v 0.04	193 ± 31	463 ± 377	2.5 ± 0.8
1000 mg/kg/day	14.2 ± 3.3	0.57 ± 0.07	214 ± 18	536 ± 295	2.9 ± 0.5

*Males*	CHO (mg/dl)	LDL (mg/dl)	HDL (mg/dl)	TG (mg/dl)	TP (g/dl)
Control	78 ± 7	6.3 ± 1.7	22.1 ± 1.4	55 ± 19	6.0 ± 0.2
250 mg/kg/day	77 ± 13	6.4 ± 2.5	22.9 ± 2.9	46 ± 16	5.9 ± 0.2
500 mg/kg/day	96 ± 19	6.8 ± 1.7	25.7 ± 3.8^b^	66 ± 29	6.0 ± 0.3
1000 mg/kg/day	121 ± 18^b^	7.7 ± 1.4	29.4 ± 3.1^b^	69 ± 29	6.3 ± 0.3^a^

*Males*	ALB (g/dl)	T-BIL (mg/dl)	A/G ratio	IP (mg/dl)	Ca (mg/dl)
Control	2.4 ± 0.1	0.07 ± 0.02	0.66 ± 0.03	9.0 ± 0.8	10.4 ± 0.5
250 mg/kg/day	2.3 ± 0.1	0.06 ± 0.01	0.64 ± 0.03	9.0 ± 0.8	10.4 ± 0.4
500 mg/kg/day	2.3 ± 0.1	0.08 ± 0.03	0.64 ± 0.02	8.8 ± 0.3	10.5 ± 0.4
1000 mg/kg/day	2.5 v 0.2	0.07 ± 0.02	0.64 ± 0.03	9.2 ± 0.6	10.8 ± 0.3

*Males*	Cl (mg/dl)	Mg (mg/dl)	Na (mg/dl)	K (mg/dl)
Control	107 ± 2	2.8 ± 0.2	146 ± 1	5.2 ± 1.1
250 mg/kg/day	107 ± 2	2.8 ± 0.3	145 ± 1	4.9 ± 0.4
500 mg/kg/day	106 ± 1	2.8 ± 0.3	146 ± 2	5.0 ± 0.6
1000 mg/kg/day	106 ± 2	2.9 ± 0.3	145 ± 1	5.0 ± 0.5

*Female*	AST (UI/L)	ALT (IU/L)	ALP (IU/L)	GGT (IU/L)	LDH(IU/L)
Control	112 ± 39	27 ± 6	167 ± 69	0.7	1235 ± 694
250 mg/kg/day	118 ± 37	33 ± 16	220 ± 75	0.8 ± 0.4	116 ± 662
500 mg/kg/day	109 ± 33	33 ± 9	215 ± 70	0.6 ± 0.5	987 ± 867
1000 mg/kg/day	105 ± 37	31 ± 7	187 ± 63	0.9 ± 0.3	1087 ± 995

*Female*	BUN (mg/dl)	CRE (mg/dl)	GLU (mg/dl)	CPK (U/L)	UA (mg/dl)
Control	16.0 ± 2.5	0.58 ± 0.08	170 ± 22	540 ± 304	0.5
250 mg/kg/day	16.7 ± 2.9	0.61 ± 0.10	166 ± 31	500 ± 206	2.5 ± 0.5
500 mg/kg/day	16.3 ± 1.8	0.58 ± 0.05	173 ± 37	399 ± 281	2.5 ± 0.7
1000 mg/kg/day	16.9 ± 2.6	0.57 ± 0.05	170 ± 20	457 ± 378	2.3 ± 0.4

*Female*	CHO (mg/dl)	LDL (mg/dl)	HDL (mg/dl)	TG (mg/dl)	TP (g/dl)
Control	79 ± 20	3.1 ± 1.0	24.0 ± 4.5	50 ± 30	6.5 ± 0.4
250 mg/kg/day	83 ± 19	3.9 ± 0.9	25.4 ± 4.3	39 ± 25	6.3 ± 0.5
500 mg/kg/day	96 ± 22	4.5 ± 1.3^a^	28.0 ± 5.0	60 ± 33	6.7 ± 0.4
1000 mg/kg/day	105 ± 14^a^	4.3 ± 0.9^a^	30.2 ± 3.3^a^	41 ± 28	6.6 ± 0.3

*Female*	ALB (g/dl)	T-BIL (mg/dl)	A/G ratio	IP (mg/dl)	Ca (mg/dl)
Control	2.9 ± 0.2	0.08 ± 0.02	0.79 ± 0.05	7.8 ± 0.5	10.6 ± 0.8
250 mg/kg/day	2.7 ± 0.2	0.09 ± 0.02	0.76 ± 0.07	8.1 ± 1.1	11.2 ± 1.7
500 mg/kg/day	0.3	0.10 ± 0.03	0.79 ± 0.08	8.3 ± 1.0	11.1 ± 0.7
1000 mg/kg/day	2.9 ± 0.2	0.09 ± 0.03	0.78 ± 0.06	8.1 ± 0.7	10.9 ± 0.6

*Female*	Cl (mg/dl)	Mg (mg/dl)	Na (mg/dl)	K (mg/dl)
Control	107 ± 2	2.6 ± 0.3	143 ± 1	5.0 ± 0.7
250 mg/kg/day	108 ± 2	2.7 ± 0.2	143 ± 1	5.3 ± 0.6
500 mg/kg/day	107 ± 2	2.6 ± 0.2	143 ± 1	5.5 ± 0.7
1000 mg/kg/day	107 ± 1	2.6 ± 0.2	143 ± 1	5.2 ± 0.7

Abbreviations: AST = Aspartate aminotransferase; AST = Aspartate aminotransferase, ALT = Alanine aminotransferase, ALP = Alkaline phosphatase, GGT = Gamma(γ)-glutamyl transferase, LDH = Lactate dehydrogenase, BUN = Blood urea nitrogen, CRE = Creatinine, GLU = Glucose, CPK = Creatine phosphokinase, UA = Uric acid, CHO = Total cholesterol, LDL = Low density lipid, HDL=High density lipid; TG = Triglyceride, TP = Total protein, ALB = Albumin, T-BIL = Total bilirubin, A/G ratio = Albumin/Globulin ratio, IP = Inorganic phosphorus, Ca = Calcium, Cl = Chloride, Mg = Magnesium, Na = Sodium, K = Potassium.

Values are Mean ± SD.

a Significant difference compare with control value p < 0.05.

b Significant difference compare with control value, p < 0.01.

**Table 8:** Summary of mean organ weight (g) and organ weight relative to body weight (%) for male in the 13 weeks oral toxicity study.

		Control (G1)	250 mg/kg/day (G2)	500 mg/kg/day (G3)	1000 mg/kg/day (G4)
*Males*					
Body Weight	g	640.31 ± 62.52	582.92 ± 53.51	595.07 ± 56.88	631.42 ± 63.42
Testis (Lt.)	g	1.79 ± 0.13	1.69 ± 0.11	1.59 ± 0.10^b^	1.76 ± 0.07
	%	0.28 ± 0.03	0.29 ± 0.03	0.27 ± 0.03	0.28 ± 0.02
Testis (Rt.)	g	1.78 ± 0.12	1.69 ± 0.13	1.60 ± 0.10^b^	1.77 ± 0.09
	%	0.28 ± 0.03	0.29 ± 0.02	0.27 ± 0.03	0.28 ± 0.03
Prostate	g	0.87 ± 0.17	0.93 ± 00.12	0.86 ± 0.14	0.82 ± 0.22
	%	0.13 ± 0.02	0.16 ± 0.02	0.14 ± 0.02	0.13 ± 0.03
Spleen	g	0.81 ± 0.10	0.81 ± 0.11	0.87 ± 0.12	0.86 ± 0.11
	%	0.12 ± 0.01	0.13 ± 0.01	0.14 ± 0.01	0.13 ± 0.02
Liver	g	17.90 ± 1.84	15.44 ± 1.84	16.54 ± 2.11	17.99 ± 1.72
	%	2.79 ± 0.13	2.64 ± 0.17	2.77 ± 0.16	2.85 ± 0.15
Adrenal (Lt.)	g	0.027 ± 0.005	0.027 ± 0.003	0.029 ± 0.004	0.027 ± 0.002
	%	0.004 ± 0.001	0.004 ± 0.0006	0.005 ± 0.0008	0.004 ± 0.0006
Adrenal (Rt.)	g	0.025 ± 0.005	0.024 ± 0.004	0.029 ± 0.005	0.026 ± 0.003
	%	0.004 ± 0.001	0.004 ± 0.0008	0.005 ± 0.0008	0.004 ± 0.0006
Kidney (Lt.)	g	1.60 ± 0.14	1.50 ± 0.20	1.63 ± 0.17	1.60 ± 0.11
	%	0.25 ± 0.01	0.25 ± 0.02	0.27 ± 0.02	0.25 ± 0.02
Kidney (Rt.)	g	1.63 ± 0.14	1.56 ± 00.24	1.63 ± 0.13	1.66 ± 0.14
	%	0.25 ± 0.01	0.26 ± 0.03	0.27 ± 0.01	0.26 ± 0.03
Heart	g	1.62 ± 0.15	1.52 ± 0.17	1.55 ± 0.10	1.69 ± 0.11
	%	0.25 ± 0.01	0.26 ± 0.01	0.26 ± 0.01	0.26 ± 0.02
Lung	g	1.74 ± 0.20	1.74 ± 0.37	1.77 ± 0.20	1.88 ± 0.29
	%	0.27 ± 0.02	0.29 ± 0.05	0.29 ± 0.03	0.29 ± 0.03
Brain	g	2.13 ± 0.07	2.17 ± 0.09	2.17 ± 0.10	2.14 ± 0.09
	%	0.33 ± 0.02	0.37 ± 0.03	0.36 ± 0.03^a^	0.34 ± 0.03
Pituitary	g	0.011 ± 0.002	0.010 ± 0.002	0.009 ± 0.002	0.009 ± 0.001
	%	0.001 ± 0.0004	0.001 ± 0.0004	0.001 ± 0.0004	0.001 ± 0.0003
Thymus	g	0.25 ± 0.09	0.30 ± 0.09	0.27 ± 0.07	0.33 ± 0.10
	%	0.04 ± 0.01	0.05 ± 0.01	0.04 ± 0.01	0.05 ± 0.01

Values are Mean ± SD.

a Significant difference compared with control, 500, 1000 mg/kg group value, p < 0.05.

b Significant difference compared with control, 500 mg/kg group value, p < 0.05.

**Table 9:** Summary of mean organ weight (g) and organ weight relative to body weight (%) for female in the 13 weeks oral toxicity study.

		Control (G1)	250 mg/kg/day (G2)	500 mg/kg/day (G3)	1000 mg/kg/day (G4)
*Females*					
Body Weight	g	325.21 ± 25.90	295.86 ± 34.91^b^	313.71 ± 14.59	286.54 ± 24.63^b^
Ovary (Lt.)	g	0.04 ± 0.009	0.04 ± 0.01	0.04 ± 0.01	0.04 ± 0.009
	%	0.012 ± 0.003	0.014 ± 0.005	0.014 ± 0.003	0.016 ± 0.003
Ovary (Rt.)	g	0.04 ± 0.01	0.04 ± 0.01	0.04 ± 0.01	0.04 ± 0.007
	%	0.012 ± 0.003	0.015 ± 0.003	0.015 ± 0.004	0.015 ± 0.002
Uterus	g	0.68 ± 0.26	0.74 ± 0.38	0.67 ± 0.33	0.71 ± 0.19
	%	0.21 ± 0.08	0.26 ± 0.16	0.21 ± 0.11	0.25 ± 0.07
Spleen	g	0.56 ± 0.06	0.57 ± 0.15	0.56 ± 0.07	0.56 ± 0.06
	%	0.17 ± 0.02	0.19 ± 0.07	0.18 ± 0.02	0.19 ± 0.01
Liver	g	8.67 ± 1.22	8.08 ± 0.92	8.49 ± 0.54	7.98 ± 1.01
	%	2.66 ± 0.31	2.75 ± 0.39	2.70 ± 0.10	2.77 ± 0.15
Adrenal (Lt.)	g	0.03 ± 0.004	0.03 ± 0.01	0.03 ± 0.005	0.03 ± 0.004
	%	0.010 ± 0.001	0.012 ± 0.003	0.010 ± 0.001	0.011 ± 0.001
Adrenal (Rt.)	g	0.03 ± 0.005	0.03 ± 0.007	0.03 ± 0.004	0.03 ± 0.004
	%	0.009 ± 0.002	0.012 ± 0.002	0.010 ± 0.001	0.010 ± 0.001
Kidney (Lt.)	g	0.93 ± 0.09	0.92 ± 0.13	0.92 ± 0.07	0.91 ± 0.12
	%	0.28 ± 0.02	0.31 ± 0.05	0.29 ± 0.02	0.32 ± 0.02
Kidney (Rt.)	g	0.97 ± 0.09	0.93 ± 0.14	0.96 ± 0.07	0.95 ± 0.16
	%	0.29 ± 0.02	0.31 ± 0.05	0.30 ± 0.02	0.33 ± 0.04
Heart	g	1.02 ± 0.09	0.92 ± 0.11^a^	1.01 ± 0.07	0.94 ± 0.07
	%	0.31 ± 0.02	0.31 ± 0.03	0.32 ± 0.01	0.33 ± 0.01
Lung	g	1.31 ± 0.13	1.27 ± 0.15	1.29 ± 0.16	1.21 ± 0.10
	%	0.40 ± 0.02	0.43 ± 0.06	0.41 ± 0.06	0.42 ± 0.03
Brain	g	1.95 ± 0.06	1.94 ± 0.10	1.96 ± 0.07	1.98 ± 0.08
	%	0.60 ± 0.05	0.66 ± 0.09	0.62 ± 0.04	0.69 ± 0.06^a^
Pituitary	g	0.016 ± 0.006	0.014 ± 0.004	0.015 ± 0.003	0.014 ± 0.003
	%	0.0050 ± 0.002	0.0049 ± 0.001	0.0048 ± 0.001	0.0051 ± 0.001
Thymus	g	0.23 ± 0.03	0.25 ± 0.04	0.26 ± 0.05	0.25 ± 0.07
	%	0.07 ± 0.006	0.08 ± 0.010	0.08 ± 0.019	0.08 ± 0.022

Values are Mean ± SD.

a Significant difference compared with 2000 mg/kg group value, p < 0.05.

b Significant difference compared with control group value, p < 0.05.

In the 90-day sub-chronic oral toxicity study, no toxicologically significant differences between the treated groups (250, 500 and 1000 mg/kg/day) and the controls were observed with respect to food consumption, ophthalmoscopy, urinalysis, haematology or clinical chemistry.

The CHO, HDL value of the males and females in the group receiving 1000 mg/kg/day Xanthigen^®^ were more elevated (p < 0.01) than in the controls. Furthermore, the HDL value of the male group receiving 500 mg/kg group (p < 0.05) and the LDL value of the female groups receiving 500 and 1000 mg/kg group (p < 0.05) were significantly higher than those of control groups (Table 7). But these changes were not considered to be toxicologically significant as there was no correlation with histopathological findings or other biochemical liver values (Table 4).

Gross pathological evaluation at the end of the study revealed vacuolization at the mid-zonal and periportal of the liver in all groups and the incidence of dosing in Xanthigen^®^ treated groups was lower than in control group. Blood exudate-filled mass at the left external acoustic pore in males treated with 250 mg/kg Xanthigen^®^ and subcutaneous mass at the right inguinal and neck region in females treated with 250 mg/kg Xanthigen^®^ were considered to be auricular chondropathy and adenocarcinoma. No treatment-related histopathological or necropsy findings were noted upon examination of the animals at the end of the study.

With regard to organ weights, the absolute liver weights of the male group treated with 250 mg/kg Xanthigen^®^, the absolute weight of both testis in males treated with 500 mg/kg Xanthigen^®^ and the absolute heart weight of the female group treated with 250 mg/kg Xanthigen^®^ were significantly lower than those of the corresponding controls (p < 0.05). Relative brain weights in males treated with 250 and 500 mg/kg Xanthigen^®^ and in females treated with 1000 mg/kg Xanthigen^®^ were significantly higher than those of the controls (p < 0.05) (results are shown in Table 8, Table 9). However, these changes were not considered to be related to the test substance as there was no correlation between the male and female groups and no dose-dependent relationship.

## 4 Discusion

There are few published reports on the safety of the two components of Xanthigen^®^. In a recent review, D´Orazio et al. [15] reported no findings in single and repeated dose studies in mice with fucoxanthin (up to a maximum of 2000 mg/kg), in clinical, laboratory or histology parameters. Another toxicology study on brown seaweeds (standardized to fucoidan) did not identify any differences between groups regarding body weight, ophthalmoscopy, urinalysis, haematology or histopathology (1350 mg/kg for 4 weeks) [16]. Meerts et al. (2009) [8] evaluated the toxicology and safety of pomegranate seed extract *in vitro* (with Ames and chromosome aberration tests) and *in vivo* (with acute toxicity and 28-day toxicity studies in Wistar rats). They reported increased hepatic enzyme levels in plasma and increased liver-to-body weight ratios in the high-dose group (13,710-14,214 mg/kg for 4 weeks), indicating no negative effects at 4.3 g/kg (the total PSO content in the daily Xanthigen^®^ dose is 300 mg).

To our knowledge, this study is the most comprehensive non-human toxicological assessment of both components combined in Xanthigen^®^. A battery testing of in vitro and animal toxicity studies on Xanthigen^®^ did not showed evidence of toxicity of this nutraceutical combination. No evidence of genotoxic or clastogenic activity from the Ames Test, the chromosome aberration test or the *in vivo* micronucleus assay in rodents –all completed in compliance with GLP and in accordance with OECD guidelines– was shown.

Neither acute nor sub-chronic oral toxicity in rats was observed in trials with doses ranging from 250 mg to 1000 mg/kg/day of Xanthigen^®^ and no adverse effects in males or females were observed after the 13-weeks treatment period, although decreased body weight, vacuolization of the liver and increases in CHO, HDL and LDL serum levels were observed in the repeated oral dose toxicity study. Interestingly, these hypercholesteraemic and lipoprotein increasing effects have been observed not only for fucoxanthin but also for other carotenoids at relatively high doses in some toxicological studies performed in rodents, the mechanism responsible for these alterations remain to be elucidated [[17], [18], [19]].

In relation to the 14-day chronic study, a clear trend in absolute and relative organ weights to the different doses could not be established, as organ weights in laboratory animals can vary significantly, which may introduce overall noise to the statistical analysis. During a survey of toxicology studies carried out by the National Cancer Institute and the National Toxicology Program, an analysis of the organ weights (absolute and relative) of Fischer 344 rats revealed increases in absolute weights and decreases in relative weights for brain, liver, right kidney, lung, heart, thyroid, and right testis associated with age. These results suggest a “general variability trend in absolute organ weights of brain < right testis < heart < right kidney < liver < lung < thymus < thyroid” [20]. While this does not correspond precisely with the results of this study, it illustrates that many variables cannot be properly controlled in these types of investigations. Nevertheless, no abnormal lesions were observed at necropsy, obviating the need for histo-pathological examination and raising no safety concerns. No variability in organ weight was observed in the 90-day repeated dose study. Additionally, we found significant decreases in body weight in male groups treated with 250 and 500 mg/kg and female groups treated with 250 and 1000 mg/kg Xanthigen^®^ during the course of the study, findings which were attributed to the weight loss properties of Xanthigen^®^ probably via a reduction in adipose tissue fat content. Previous in vivo studies performed in mouse [21] and clinicals trial in humans [10] have indicated that Xanthigen^®^ is involved in adipose tissue fat depletion leading to weight loss. Unfortunately, this hypothesis was not able to be confirmed because adipose tissue analysis was not part of the standardized subchronic 90-day oral toxicity protocol.

Finally, changes in liver histology were not related to Xanthigen^®^ as there was no correlation with the doses used and because they were observed more frequently in the control group than in the treated groups. Furthermore, no significant increase in liver enzymes was detected. This is consistent with the published Xanthigen^®^ clinical trial that did not show any adverse effects on the liver parameters. In fact, those subjects with elevated levels of liver enzymes related to fatty liver demonstrated a significant improvement in these parameters over the course of the study.

## 5 Conclusion

Based on the observations and analysis performed reported here, it was concluded that Xanthigen^®^ did not present neither genotoxic nor clastogenic activity using the Ames test, the chromosome aberration test and in vivo micronucleus assay. No safety issues were observed in any of the doses used in the 14-day and 90-day repeated oral toxicological assay in rats. Some treatment-related changes were detected but these were not considered toxicologically significant. The NOAEL of Xanthigen^®^ for the 90-day repeated dose toxicity study was the highest dose tested 1000 mg/kg/day.

## Funding

Supported by funds from Nektium Pharma S.L. (formerly Polinat S.L.)

## Conflicts of interest

L. López-Rios, T. Vega, R. Pérez-Machín and JC Wiebe are currently employed by Nektium Pharma SL (Las Palmas, Spain) and JC Jung work for NOVAREX Co., Ltd., the two companies that funded this study.

## Transparency document

Transparency document
